# Temperature-Rise Suppression Concrete Incorporating Steel-Encapsulated SAP–Water Phase-Change Aggregates: Semi-Adiabatic Characterization, Adiabatic Temperature-Rise Prediction and Finite Element Assessment

**DOI:** 10.3390/ma19122630

**Published:** 2026-06-18

**Authors:** Heng Yin, Tianheng Yuan, Zongjin Li, Zhenzhen Yin, Hong Yao, Fuqiang Wang

**Affiliations:** 1 Department of Engineering Science, Faculty of Innovation Engineering, Macau University of Science and Technology, Avenida Wai Long, Taipa, Macao SAR 999078, China; 3230001934@student.must.edu.mo (H.Y.); 3230001822@student.must.edu.mo (T.Y.); 3230001906@student.must.edu.mo (Z.Y.); 2 Kailuan (Group) Limited Liability Corporation, Tangshan 063000, China

**Keywords:** temperature-rise suppression concrete, steel-encapsulated SAP–water aggregates, phase-change aggregates, adiabatic temperature rise, finite element simulation

## Abstract

**Highlights:**

- Steel-encapsulated SAP–water aggregates were developed for temperature-rise suppression concrete.
- Semi-adiabatic tests showed reduced peak temperature rise and delayed thermal peaks.
- Adiabatic temperature rise was reconstructed from semi-adiabatic measurements.
- FEM accurately reproduced the measured thermal evolution of the concrete mixtures.
- SAP-15 reduced pile-cap peak temperatures and mitigated thermal stress development.

**Abstract:**

Early-age temperature rise in mass concrete can generate substantial thermal gradients and increase the risk of cracking. In this study, a temperature-rise suppression concrete was developed by partially replacing conventional coarse aggregate with steel-encapsulated superabsorbent polymer (SAP)–water phase-change aggregates. Semi-adiabatic temperature-rise tests were conducted to characterize the early-age thermal response, and the corresponding adiabatic temperature-rise histories were reconstructed using a heat-loss compensation method. The results showed that the incorporation of steel-encapsulated SAP–water aggregates reduced the temperature rise and delayed the thermal peak under semi-adiabatic conditions. For SAP-15, the peak core temperature in the validated finite element simulation decreased from 51 °C to 44 °C, while the maximum adiabatic temperature rise decreased to 40.5 °C. Engineering-scale simulation of a bridge pile-cap foundation further showed reductions in internal peak temperature, temperature difference, and thermal stress. These findings demonstrate that steel-encapsulated SAP–water phase-change aggregates provide an effective material-based strategy for moderating early-age thermal accumulation and mitigating thermal cracking risk in mass concrete.

## 1. Introduction

Mass concrete structures, such as bridge pile caps, piers, and large foundations, commonly experience a substantial temperature rise during the early hydration stage [1]. Huang et al. [2] reported that the temperature of mass concrete increased to 78.0–88.3 °C within 20 h after casting, while Liu et al. [3] observed a temperature rise of 40.0–48.8 °C in a bridge pier support. Because the heat generated by cement hydration cannot be dissipated efficiently from the interior of a large concrete body, a pronounced temperature gradient may develop between the core and the surface [4]. The temperature gradients across different sections of concrete structures were discussed in the studies by Muneer Saeed and Feng Lin [5,6]. This non-uniform thermal field can induce significant restraint stresses, which in turn increase the risk of early-age cracking and compromise the long-term durability of the structure [7,8]. Therefore, effective control of temperature rise and internal thermal gradients remains a central challenge in the design and construction of mass concrete [2,9].

A variety of strategies have been developed to mitigate hydration-induced thermal accumulation, including optimization of binder composition, reduction in cement content, precooling of constituent materials, and installation of internal cooling pipes. Internal pipe cooling is a well-established engineering method for temperature control in mass concrete, and recent studies have further improved its prediction and application in pipe-cooled mass concrete structures [10]. Leng et al. [11] investigated the application of temperature inhibitors to control hydration temperature rise in concrete; Tian’s research [12] demonstrated that raw material pretreatment could effectively reduce the hydration temperature increase. Agathe Bourchy conducted experiments with varying concrete mix proportions, finding that gel-based admixtures replacing partial cement content significantly lowered hydration temperature rise [13,14,15,16,17]. Although these approaches can be effective, they often require additional construction complexity, external energy input, or compromises in early-age performance. In this context, material-based thermal regulation has attracted increasing attention as a more intrinsic and potentially more practical route for controlling the early-age thermal response of concrete [18,19]. Among the candidate solutions, phase-change materials (PCMs) are particularly attractive because they can absorb and release latent heat during phase transition, thereby buffering temperature fluctuations within the host matrix [20,21,22]. Fabio Fernandes [23] investigated how phase-change microcapsules incorporated into concrete significantly altered the hydration rate and temperature of the material. Pang’s research [24] demonstrated that phase-change aggregate concrete exhibits outstanding energy storage performance. Wang et al. [25] conducted experimental studies to evaluate the effects of PCM addition on the exothermic hydration process and mechanical properties of concrete.

Despite this promise, the direct incorporation of PCMs into cementitious materials remains challenging. Conventional PCM systems may suffer from leakage, insufficient mechanical robustness, incompatibility with the cement matrix, or limited suitability for structural concrete applications [26]. Khaled Own Mohaisen’s research [27] demonstrated that increased PCM incorporation significantly reduced concrete compressive strength, with phase-change material leakage observed during experiments. In Pejman Keikhaei Dehdezi’s study [28], the compressive strength decreased from 52 MPa to 9.7 MPa when the PCM content increased from 0% to 5%, where 5% PCM indicated that the mass of PCM replaced an equivalent 5% of the concrete mass. Li’s research [29] confirmed PCM’s energy storage benefits in lightweight concrete applications requiring lower strength requirements. To address these limitations, this study proposes a novel aggregate-scale thermal regulation strategy based on steel-encapsulated superabsorbent polymer (SAP)–water phase-change aggregates [30]. In this system, the phase-change medium is confined within hollow steel shells that function as artificial coarse aggregates. Such a design aims to combine the latent-heat storage capacity of the internal phase-change medium with the mechanical load-bearing role of coarse aggregate, thereby enabling multifunctional performance in concrete.

On this basis, a temperature-rise suppression concrete was developed by partially replacing natural coarse aggregates with steel-encapsulated SAP–water phase-change aggregates. The thermal behavior of the resulting concrete was first characterized through semi-adiabatic temperature-rise tests, and the corresponding adiabatic temperature-rise histories were subsequently reconstructed using a heat-loss compensation method. A finite element model was then established and calibrated against the experimental semi-adiabatic temperature-rise data. Finally, the validated model was extended to the simulation of a practical bridge pile-cap foundation to evaluate the engineering-scale effectiveness of the proposed concrete system. The results show that the incorporation of steel-encapsulated SAP–water aggregates reduced the maximum temperature rise, delayed the thermal peak, and alleviated the temperature difference and thermal stress in the simulated mass concrete structure.

Therefore, the core objective of this study is to establish a material-based approach for suppressing early-age temperature rise in mass concrete while maintaining structural applicability. The novelty of this work lies in integrating macro-encapsulated phase-change functionality into aggregate design, combining semi-adiabatic testing with adiabatic temperature-rise prediction, and further linking laboratory-scale thermal characterization to engineering-scale finite element assessment. This study is expected to provide both a new material concept and a quantitative evaluation framework for the design of crack-resistant mass concrete.

## 2. Experiment Details

### 2.1. Raw Materials

The binder used was Portland cement (P•O 42.5, corresponding to strength class 42.5 MPa per Chinese standard GB 175-2023), supplied by Jidong Heidelberg (Jingyang) Cement Co., Ltd. (Jingyang, Shanxi, China). Its chemical composition is provided in Table 1. A polycarboxylate-based high-performance superplasticizer manufactured by Jiana Company was employed. The fine aggregate was natural river sand, with a saturated-surface-dry (SSD) water absorption of 1% and an apparent density of 2559.52 kg/m^3^. The coarse aggregate consisted of crushed limestone with a continuous gradation of 5–40 mm, exhibiting an SSD water absorption of 1.3% and an apparent density of 2682.68 kg/m^3^.

### 2.2. Fabrication of the Steel-Encapsulated SAP–Water Aggregates

A multifunctional artificial phase-change aggregate was designed and fabricated, integrating a structural skeleton with phase-change material encapsulation functionality. The aggregate uses a hollow cast-iron sphere as the carrier, featuring an outer diameter of 30 mm, a wall thickness of 1.8 mm, and an apparent density of approximately 2264 kg/m^3^. Six evenly distributed holes, each 2 mm in diameter, were drilled on the sphere’s surface to enable phase-change material filling. A superabsorbent polymer (SAP) with high water absorption capacity was selected as the filling medium.

**Figure 1:**
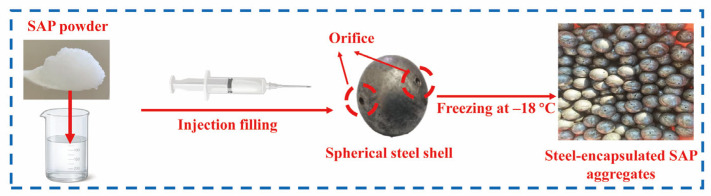
Preparation process of the Steel-encapsulated SAP–water aggregates.

The SAP–water phase-change aggregates were prepared using a pressure injection method. SAP powder was first mixed with water at a mass ratio of 1:60 to form a hydrated gel, which was then injected into the hollow steel spheres using a syringe until the gel overflowed from the remaining holes, indicating complete and dense filling. No additional chemical sealant was applied to the drilled holes. Instead, during pressure injection, the hydrated SAP–water gel was compressed and deformed while passing through the holes and was mechanically retained inside the steel shell after entering the cavity. After the injection pressure was released, the swollen SAP–water gel did not naturally flow out through the small holes because of its gel state and limited flowability. The mass of each aggregate increased by approximately 8 g after filling, and no visible leakage was observed. To evaluate leakage resistance during mixing, 15 filled aggregates were mixed in cement mortar with a water-to-cement ratio of 0.47 for 2 min. As summarized in Table 2, the average mass changed only from 42.21 g to 42.13 g, corresponding to a mass change rate of 0.18%. This result confirms the mass stability and leakage resistance of the steel-encapsulated SAP–water aggregates under the mixing condition. The prepared aggregates were subsequently frozen at −18 °C for 24 h and then stored at −18 °C before concrete mixing. During concrete preparation, the frozen aggregates were taken out immediately before use and added during the final mixing stage to minimize temperature recovery before casting (see Figure 1).

In this study, SAP was used as a water-retaining carrier rather than as an intrinsic phase-change material. The actual phase-change medium was the absorbed water retained in the SAP gel. Therefore, the relevant phase transition of the proposed aggregate system was the freezing–melting transition of water, occurring near 0 °C under normal pressure. The use of SAP was intended to immobilize water inside the steel shell and reduce the risk of free-water release during melting. This design differs from conventional internal curing using directly dispersed SAP particles; here, the SAP–water gel was confined inside hollow steel shells to form an aggregate-scale thermal-buffering inclusion. The absorbed water provides sensible heat storage and latent heat absorption during melting, while the steel shell provides geometric confinement and leakage resistance.

### 2.3. Formulation and Mixture of the Temperature-Rise Suppression Concrete

The steel-encapsulated SAP–water phase-change aggregates were incorporated into the concrete by replacing the coarse aggregate at an equivalent volume. The mixed proportions of each specimen are presented in Table 3. The specimen nomenclature was defined as follows: SAP-5 denotes concrete containing phase-change aggregates with a diameter of 30 mm at a volume fraction of 5% as a replacement for coarse aggregate, while SAP-10 denotes concrete containing phase-change aggregates with a diameter of 30 mm at a volume fraction of 10% as a replacement for coarse aggregate. The other specimens were named according to the same convention. During sample preparation, the coarse and fine aggregates were first mixed in a mixer for approximately 60 s, followed by the addition of cement and further mixing for approximately 30 s. Water and superplasticizer were then premixed and added to the mixer, and the mixture was stirred for 60 s. Subsequently, the phase-change aggregates were introduced and mixed for an additional 30 s to ensure their uniform distribution. After mixing, the slump of the fresh concrete was measured to be 130–150 mm, meeting the required range. The concrete was then immediately transferred into the insulated test chamber, compacted by vibration, and subjected to semi-adiabatic temperature-rise monitoring. During the experimental process, particular care was taken to keep the phase-change aggregates in a frozen state as much as possible before their addition to the mixer, and to start temperature monitoring as soon as possible after casting.

## 3. Experimental Results and Discussion

### 3.1. Semi-Adiabatic Temperature Rise Test

**Figure 2:**
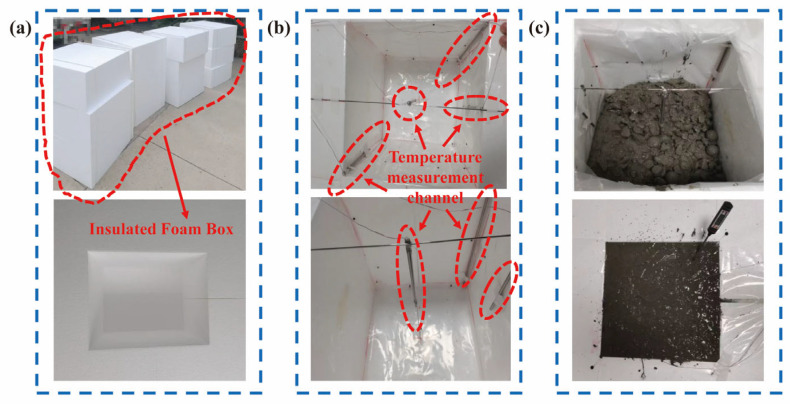
Experimental setup for semi-adiabatic temperature rise testing: (**a**) insulated testing box; (**b**) configuration of temperature sensors; (**c**) casting of the temperature-rise suppression concrete.

The test apparatus was designed based on established methodologies reported in previous studies [31,32,33], as illustrated in Figure 2. The insulation box was constructed from expanded polystyrene (EPS) foam boards with a thickness of 300 mm, a density of 18 kg/m^3^, and a thermal conductivity of 0.039 W/(m·K). The overall external dimensions of the box were 900 mm × 900 mm × 900 mm, and its interior was vertically divided into three layers, each 300 mm in height. A central cavity measuring 300 mm × 300 mm × 300 mm was formed within the middle layer to accommodate the concrete specimen for temperature monitoring. 4 temperature sensors were positioned at specific locations: the geometric center of the concrete block, the center of one side face, the midpoint of one side edge, and the center of a bottom corner [33]. The average temperature of the concrete during the test was estimated by integrating the data from these measurement points. The entire semi-adiabatic test setup was placed in an ambient environment maintained at 20–24 °C, and the temperature was continuously monitored for 7 days.

### 3.2. Semi-Adiabatic Temperature Rise Results

**Figure 3:**
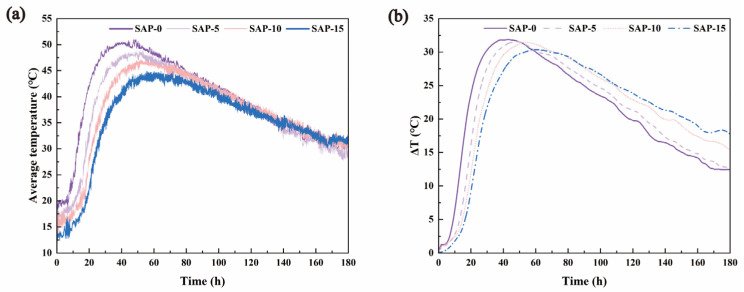
Temperature monitoring data: (**a**) mean temperature readings from sensors; (**b**) smoothed ΔT curve.

Figure 3 presents the semi-adiabatic temperature evolution of concrete incorporating steel-encapsulated SAP–water aggregates alongside a control specimen prepared with natural coarse aggregate. All mixtures exhibited typical hydration heat evolution curves, characterized by a rapid temperature increase at early ages, followed by a gradual decline as the hydration rate diminished [33,34,35,36]. Notably, the incorporation of steel-encapsulated SAP–water aggregates significantly reduced heat accumulation, confirming their effectiveness in suppressing temperature rise under semi-adiabatic conditions. The control mixture exhibited the highest temperature rise, reaching a peak relative temperature increase of 31.86 °C at 43.31 h. In contrast, replacing natural coarse aggregate with steel-encapsulated SAP–water aggregates reduced the magnitude of the peak temperature rise and delayed its occurrence. Specifically, the SAP-5 mixture attained a peak relative temperature increase of 31.56 °C at 48.18 h, corresponding to a delay of approximately 4.88 h relative to the control. This delay became more pronounced with increasing replacement ratios. The SAP-15 mixture showed the most substantial delay, peaking at 30.40 °C at 58.44 h—approximately 15.13 h later than the control.

In addition to delaying the temperature peak, steel-encapsulated SAP–water aggregates also reduced the maximum temperature rise. Compared to the control, the SAP-15 mixture lowered the peak relative temperature rise by 1.46 °C. The thermal regulation capability of steel-encapsulated SAP–water aggregates depends on the volume fraction introduced into the concrete system. This behavior aligns with the design mechanism of the macro-encapsulated water–ice phase-change system, which absorbs heat through latent heat of fusion during the temperature-rise stage, thereby limiting heat accumulation and effectively suppressing the overall temperature increase.

The proposed system differs from conventional pre-cooling methods and the use of ice as a mixing water. Pre-cooling or partially freezing ordinary aggregates can reduce the initial temperature of fresh concrete; however, mineral aggregates have a relatively limited heat-storage capacity compared with water. In the present design, the absorbed water retained by SAP provides both sensible heat storage and latent heat absorption during melting, allowing the inclusions to buffer the temperature rise over a longer period of early hydration. Compared with replacing part of the mixing water with ice, the SAP gel confines the absorbed water and reduces the risk of releasing additional free water into the cementitious matrix during melting. Therefore, the main function of the steel-encapsulated SAP–water aggregate is not only to lower the initial casting temperature, but also to provide a controlled, aggregate-scale thermal buffer without directly disturbing the designed water-to-cement ratio.

In summary, the semi-adiabatic calorimetry results confirm that the proposed steel-encapsulated SAP–water aggregates offer a practical and effective approach to hydration heat management in concrete. By simultaneously reducing the maximum temperature rise and delaying the temperature peak, this strategy demonstrates significant potential for mitigating thermal cracking risks in mass concrete structures and contributes to the development of thermally adaptive and sustainable cement-based infrastructure [37,38].

## 4. Simulation of Practical Project

### 4.1. Calculation of the Adiabatic Temperature Rise

In previous studies, the semi-adiabatic temperature rise test has been demonstrated to be a highly accurate method for predicting the adiabatic temperature rise in concrete [39]. In particular, the application of a heat loss compensation method to correct semi-adiabatic temperature rise curves and obtain adiabatic curves has been widely adopted [40,41].

The deviation between adiabatic and semi-adiabatic measurements stems from partial heat loss to the surroundings [42]. By accounting for this lost heat, the adiabatic temperature rise can be estimated from semi-adiabatic test data. This relationship can be expressed as

(1) $$T_{g}=T_{v}-T_{p}+\lambda \int_{0}^{t}T_{s}(t)-T_{a}(t)dt$$

where $T_{g}$ denotes the adiabatic temperature rise, $T_{v}$ represents the average temperature of the specimen, λ is the heat loss coefficient of the semi-adiabatic apparatus, $T_{s}$ is the surface temperature of the specimen, $T_{a}$ is the ambient temperature.

The average temperature $T_{v}$ and surface temperature $T_{s}$ of the specimen are defined as

(2) $$\begin{array}{l} T_{v}=\frac{1}{V}\int T(x,y,z)dV\\ T_{s}=\frac{1}{A}\int T(x,y,z)dA \end{array}$$

The temperature distribution within the concrete specimen can be approximated by a fourth-order polynomial:

(3) $$T(x,y,z)=\omega_{1}+\omega_{2}(x^{4}+y^{4}+z^{4})+\omega_{3}(x^{4}y^{4}+y^{4}z^{4}+z^{4}x^{4})+\omega_{4}(x^{4}y^{4}z^{4})$$

where $\omega_{1}$ to $\omega_{4}$ are undetermined coefficients. In normalized coordinates, the temperatures at four specific locations are measured: geometric center $\left(0,0,0\right)$, $T_{m}$; center of one face $\left(0,1,0\right)$, $T_{f}$; midpoint of one edge $\left(1,1,0\right)$, $T_{e}$; one corner $\left(1,1,1\right)$, $T_{c}$.

Substituting Equation (3) into Equation (2) and integrating yields the coefficients $\omega_{1}$ to $\omega_{4}$, leading to the following explicit expressions for $T_{v}$ and $T_{s}$:

(4) $$\begin{array}{l} T_{v}=\frac{64}{125}T_{m}+\frac{48}{125}T_{f}+\frac{12}{125}T_{e}+\frac{1}{125}T_{c}\\ T_{s}=\frac{16}{25}T_{f}+\frac{8}{25}T_{e}+\frac{1}{25}T_{c} \end{array}$$

Thus, by monitoring the temperatures at the four locations (center, face, edge, and corner) within the semi-adiabatic setup, the average and surface temperatures of the specimen can be estimated at any time during the test.

The heat loss coefficient is assumed to be independent of ambient and internal temperatures. It can be determined during the stage when cement hydration is essentially complete. Differentiating Equation (1) with respect to time gives

(5) $$\frac{\partial T_{g}}{\partial t}=\frac{\partial T_{v}}{\partial t}+\lambda (T_{s}-T_{a})$$

When hydration is nearly complete, $T_{g}$ stabilizes, reducing Equation (5) to

(6) $$\lambda =-\frac{\partial T_{v}}{(T_{s}-T_{a})\partial t}$$

In this study, the period between 120 h and 170 h after concrete casting was used to estimate λ for each of the four semi-adiabatic apparatuses. The obtained values were

$$\begin{array}{l} \lambda_{1}=0.00996\\ \lambda_{2}=0.00984\\ \lambda_{3}=0.00972\\ \lambda_{4}=0.00873 \end{array}$$

**Figure 4:**
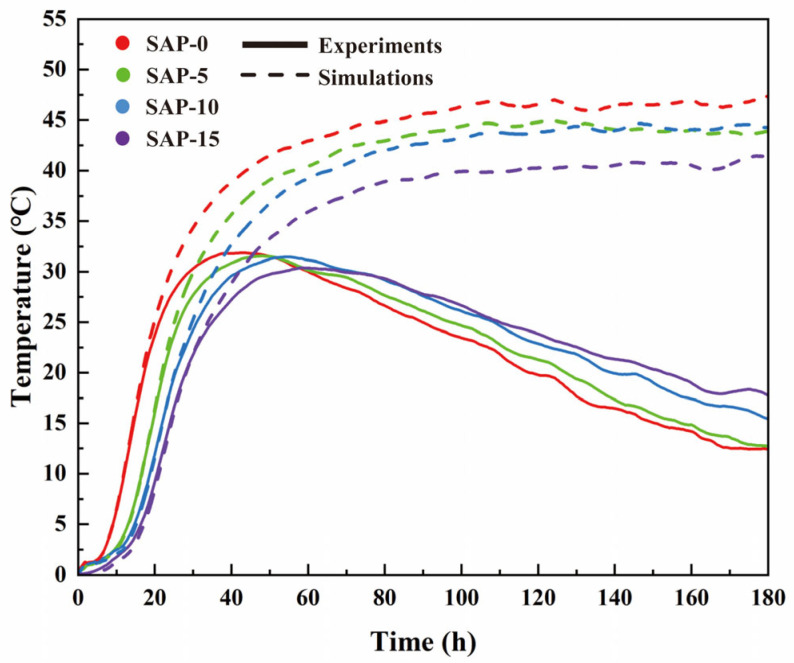
Prediction of adiabatic temperature rise for the temperature-rise suppression concrete.

Using the above framework, the adiabatic temperature rise $T_{g}$ was predicted from the semi-adiabatic measurements for all four concrete mixtures throughout the test duration, and the result is shown in Figure 4.

Based on the semi-adiabatic temperature rise tests and the simulated hydration heat release under equivalent adiabatic conditions, Figure 4 clearly reveals the significant regulatory effect of steel-encapsulated SAP–water aggregates on the early-age thermal evolution of concrete. The control group (SAP-0) exhibited typical hydration characteristics of mass concrete, with temperature rising rapidly over time and reaching its peak adiabatic temperature rise at approximately 90 h. This group showed the highest peak amplitude, reflecting a substantial accumulation of cement hydration heat within the system. In contrast, the incorporation of phase-change aggregates markedly altered the overall temperature-rise curve: on one hand, the maximum temperature rise was significantly reduced; on the other hand, the time to reach the peak temperature was delayed, indicating that both the heat-release rate and the thermal accumulation process were effectively suppressed.

Specifically, with increasing SAP content (SAP-5, SAP-10, SAP-15), the slope of the temperature-rise curve during the early stage decreased slightly, demonstrating that the phase-change aggregates began to exert a “thermal buffering” effect even at the onset of hydration. Among these, the SAP-5 and SAP-15 groups showed particularly pronounced reductions in peak temperature rise, with maximum adiabatic temperature rises substantially lower than that of the control group. Meanwhile, the delayed and flattened temperature peak confirms that latent-heat absorption during the ice-to-water phase transition effectively buffered the heat generated by cement hydration. This phenomenon indicates that the macro-encapsulated phase-change material not only lowered the thermal peak of the system but also, by delaying the concentrated release of heat, significantly improved the development of the internal temperature gradient in the concrete.

Overall, the figure provides visual evidence of the “dual regulatory effect” of Steel-encapsulated SAP–water aggregates on the temperature evolution of concrete under semi-adiabatic and equivalent adiabatic conditions—namely, reducing the maximum temperature rise and reshaping the heat-release history. This intrinsic thermal regulation mechanism offers a reliable material-science basis for mitigating temperature-induced stresses and early-age cracking risks in mass or highly restrained concrete elements, and it also establishes an experimental foundation for subsequent large-scale thermal-mechanical coupled finite-element analyses.

### 4.2. Validation of the Finite Element Model Against Experimental Semi-Adiabatic Temperature Rise Data

**Figure 5:**
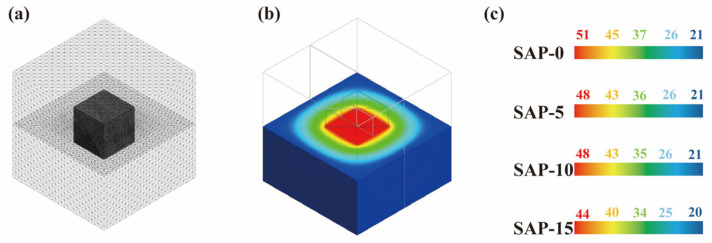
Finite element analysis of semi-adiabatic temperature rise and simulated temperature distribution: (**a**) FEA modeling setup; (**b**) typical temperature contour in the 30 cm cube specimen; (**c**) simulated temperature distributions of SAP-0, SAP-5, SAP-10, and SAP-15 at their respective peak-temperature times after casting, approximately 43 h, 48 h, 52 h, and 58 h.

A finite element analysis model for simulating the semi-adiabatic temperature rise was established using MIDAS FEA NX 2024 R1, as shown in Figure 5a. The model consists of a dark-colored cubic core representing the 30 cm × 30 cm × 30 cm concrete specimen, surrounded by a 30 cm-thick layer of expanded polystyrene (EPS) foam illustrated by the light-gray mesh. The model dimensions correspond exactly to those used in the actual semi-adiabatic test. The EPS foam was assigned a density of 18 kg/m^3^, a thermal conductivity of 0.039 W/(m·K), and a specific heat capacity of 1300 J/(kg·K). The heat generation due to cement hydration was defined by the adiabatic temperature-rise curve derived in the preceding section, which was input into the model to perform the semi-adiabatic temperature-rise simulation.

Figure 5b presents a typical contour plot of the temperature distribution inside the concrete specimen. The temperature field was interpreted according to the color scale shown in the corresponding legend. The red region corresponds to the maximum temperature of approximately 51 °C, whereas the blue region corresponds to the minimum temperature of approximately 20 °C. The concrete core exhibits the highest temperature due to accumulated hydration heat, and the temperature gradually decreases from the core toward the surrounding insulation layer.

**Figure 6:**
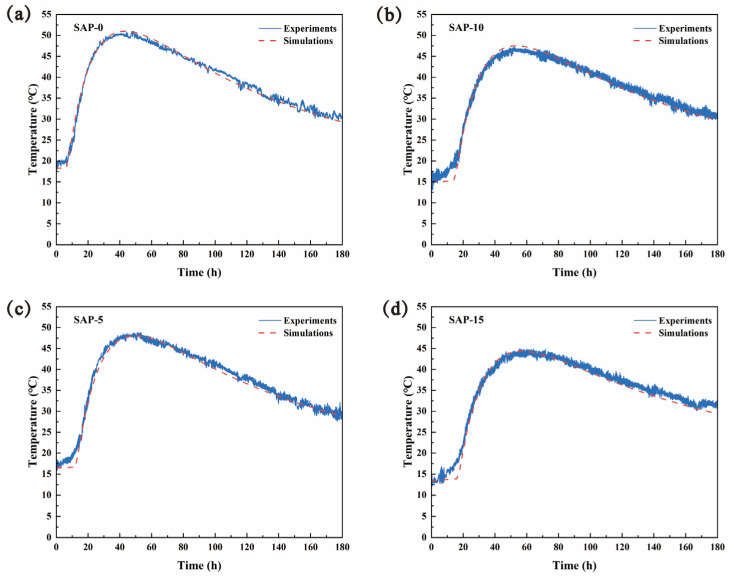
Optimal finite element model identified from simulations of four semi-adiabatic temperature rise tests: (**a**) SAP-0; (**b**) SAP-10; (**c**) SAP-5; (**d**) SAP-15.

Figure 5c illustrates the simulated temperature distributions of the four mixtures at their respective peak-temperature times after casting. According to the temperature-history curves in Figure 6, the temperature fields were evaluated at approximately 43 h, 48 h, 52 h, and 58 h for SAP-0, SAP-5, SAP-10, and SAP-15, respectively. The results show that the peak temperature in the SAP-0 specimen reached 51 °C, with heat diffusing outward from the center and temperatures gradually decreasing towards the exterior. The temperature of the expanded polystyrene (EPS) foam in direct contact with the concrete reached 45 °C, while the outer surface of the insulation foam remained near the ambient temperature of 21 °C. The temperature profiles of SAP-5 and SAP-10 were similar, both exhibiting lower peak temperatures compared with SAP-0. The central peak temperature in these mixes was approximately 48 °C, and the contacting EPS foam temperature reached 43 °C. The lowest central peak temperature was observed in SAP-15, measuring only 44 °C. These results further show that the incorporation of steel-encapsulated SAP–water aggregates reduced the peak temperature rise caused by cement hydration.

The primary thermal characteristic of concrete targeted in this study is its adiabatic temperature rise. A commonly used two-parameter expression, Equation (7), was employed as the model for the adiabatic temperature rise [31,43]:

(7) $$Q(t)=Q_{\infty }(1-e^{\left(-rt\right)})$$

where r is the reaction factor. Using appropriate values of $Q_{\infty }$ and r, the model can accurately simulate the heat of hydration of concrete.

In the finite element model, the adiabatic temperature-rise function was used to define the time-dependent hydration heat generation of concrete. The maximum adiabatic temperature rise $Q_{\infty }$ controlled the total heat-release potential, while the reaction factor r controlled the rate of heat evolution. The heat-source parameters were calibrated by matching the simulated semi-adiabatic temperature histories with the experimental results. The calibrated parameters used in the model are summarized in Table 4, including density, thermal conductivity, specific heat capacity, reaction factor, and maximum adiabatic temperature rise.

For the remaining parameters required in the FEM thermal analysis, a sensitivity study was first conducted by testing the cooling rate of hot water inside the polystyrene foam box. This step was used to determine the thermal conductivity and specific heat of the foam insulation [40]. Subsequently, the heat-generation parameters of concrete were calibrated against the semi-adiabatic temperature-rise tests. The reaction factor r and the maximum adiabatic temperature rise $Q_{\infty }$ were used to define the time-dependent hydration heat source. The calibrated parameters for all mixtures are listed in Table 4. These parameters were determined by minimizing the difference between the experimental temperature histories and the simulated results [31].

Through this parameter optimization based on input sensitivity studies, the sum of squared errors between the semi-adiabatic test data and the simulated data was minimized for all four concrete types, achieving the best possible agreement between experiment and finite element simulation [44]. The calibrated model can therefore be applied to guide the construction of mass concrete engineering projects. A comparison between the simulated and experimentally measured results for the four semi-adiabatic temperature rise tests is presented in Figure 6.

### 4.3. Finite Element Modeling and Analysis of a Pier (Pile-Cap) Foundation

The temperature rise simulation was conducted with reference to the actual construction scheme of the main pier cap of the Guangdong Hongqili Waterway Rail-Road Bridge. The cap has plan dimensions of 55 m × 39 m and a thickness of 7 m. It was cast in three layers from bottom to top. Within the height of the cap, six layers of cooling pipes were uniformly arranged, with a horizontal spacing of 1 m. Each casting layer contained two layers of cooling pipes. During the construction period, the environmental wind speed ranged from 0.5 to 3.5 m/s, the maximum air temperature was 32 °C, and the minimum was 15 °C. Steel formwork was used for the side surfaces of the cap. After casting, the formwork and the top surface were covered with an insulating cotton blanket as thermal insulation.

**Figure 7:**
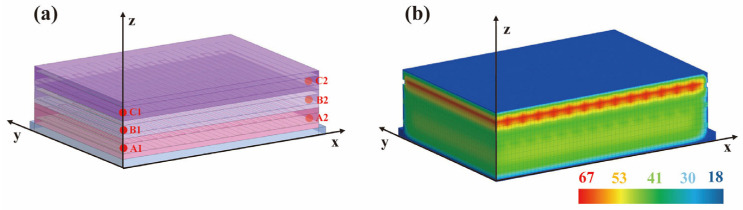
Finite element simulation of the pile-cap foundation: (**a**) structural model and arrangement of monitoring points; (**b**) representative contour plot of the simulated temperature distribution in the pile-cap foundation at a certain moment after the third-layer casting, with the color bar indicating the temperature scale.

To simplify the calculation and take advantage of structural symmetry, the model was assumed to be centrally symmetric about the *Z*-axis. Therefore, a quarter model of the original design was adopted for the numerical simulation, as illustrated in Figure 7a. For temperature and stress analysis, six monitoring points were selected: the center point of each of the three layers, and three near-surface points located 50 cm from the exterior surface (considering the influence of ambient temperature). The monitoring points were labeled according to the casting layer and location. A, B, and C denote the first, second, and third casting layers, respectively. The number “1” denotes the center point of the corresponding layer, while “2” denotes the near-surface point. Thus, A1, B1, and C1 represent the center points of Layers 1–3, respectively, and A2, B2, and C2 represent the corresponding near-surface points. Figure 7b shows a typical contour plot of the temperature distribution inside the pile-cap foundation after the third-layer casting. The temperature field was interpreted according to the color scale shown in the corresponding legend. The red region corresponds to the maximum temperature of approximately 67 °C, whereas the blue region corresponds to the minimum temperature of approximately 18 °C. The concrete core exhibits a higher temperature due to the accumulation of hydration heat, and the heat then diffuses toward the surrounding areas.

**Figure 8:**
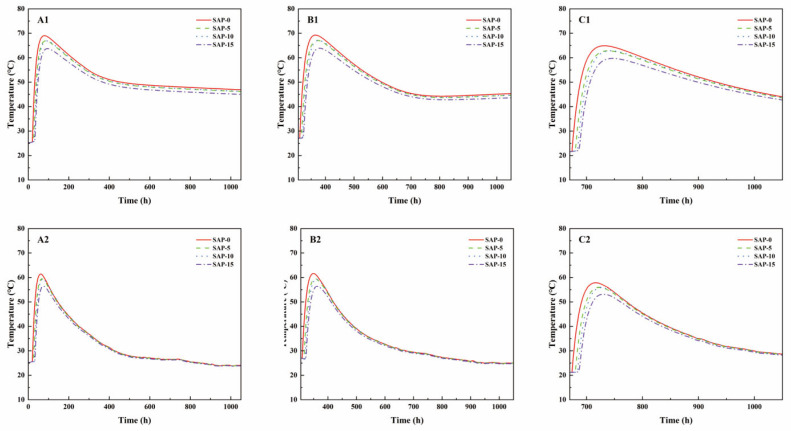
Simulated temperature evolution at the six monitoring points of the pile-cap foundation: A1, B1, and C1 represent the center points of the first, second, and third casting layers, respectively; A2, B2, and C2 represent the corresponding near-surface points.

Figure 8 presents the simulated temperature evolution curves, clearly illustrating the thermal buffering effect introduced by the steel-encapsulated SAP–water aggregates. Compared to the reference ordinary C30 concrete, mixtures incorporating steel-encapsulated SAP–water aggregates exhibited a reduction in the maximum temperature rise and a delay in the time to reach the peak temperature. The observed temperature gradient between the interior and near-surface regions is attributed to restricted heat dissipation, resulting in a higher temperature peak in the core.

A systematic increase in the content of steel-encapsulated SAP–water aggregates significantly lowered the maximum temperature and extended the time required to reach it. Notably, the SAP-15 mixture demonstrated the most pronounced suppression effect and the longest delay in temperature rise. Its temperature curve displayed a distinct plateau during the phase-change period, confirming that the latent heat absorption of the encapsulated phase-change material effectively mitigated the heat accumulation caused by cement hydration.

Furthermore, higher SAP content contributed to a more uniform thermal distribution within the temperature-rise suppression concrete, reducing the temperature difference between the interior and near-surface regions. This resulted in a more homogeneous thermal field and consequently lower thermal stress.

These results indicate that steel-encapsulated SAP–water aggregates have potential as aggregate-scale thermal-regulation inclusions in mass concrete. However, their mechanical contribution and long-term durability require further experimental validation before their structural applicability can be fully confirmed.

Table 5 summarizes the thermophysical parameters and experimentally measured elastic modulus values used in the finite element analysis. To directly compare the thermophysical properties of the reference and SAP-modified concretes, thermal conductivity was measured at 25 °C using a TPS2500S thermal constant analyzer based on the Hot Disk transient plane source method, following ISO 22007-2. Three repeated measurements were conducted for each mixture, and the average thermal conductivities of SAP-0, SAP-5, SAP-10, and SAP-15 were 1.340, 1.745, 1.650, and 1.353 W/(m·K), respectively. The elastic modulus was measured at 28 days, and three specimens were tested for each mixture. The average values were used as the macroscopic elastic parameters in the thermal-stress simulation. The measured elastic modulus values of SAP-0, SAP-5, SAP-10, and SAP-15 were 42.55, 37.8, 37.1, and 38.4 GPa, respectively. Thus, the influence of the steel shells and SAP–water inclusions was incorporated through the measured overall elastic response of each concrete mixture. In the engineering-scale model, each concrete mixture was treated as a homogenized material rather than explicitly modeling the steel shells as separate mesoscopic phases. As the content of steel-encapsulated SAP–water aggregates increased, the effective specific heat capacity increased from 0.801 to 0.912 kJ/(kg·K), while the maximum adiabatic temperature rise decreased from 46.7 °C to 40.5 °C. The non-monotonic variation in thermal conductivity indicates that the observed temperature-rise suppression cannot be explained solely by a low-thermal-conductivity effect. Instead, it is mainly associated with the increased heat-storage capacity and latent heat absorption of the frozen SAP–water system during melting.

The simulated internal peak temperatures and the maximum temperature differences between the interior and exterior surfaces for each casting layer (Layers 1 to 3) are listed in Table 6. Compared to the C30 reference group, all mixes containing steel-encapsulated SAP–water aggregates demonstrated significant temperature-rise suppression. For instance, in Layer 1, the internal peak temperature of the mix with 15% SAP was 5.3 °C lower than that of C30 (representing a reduction of approximately 7.7%), and the temperature difference between the interior and exterior surfaces decreased by 1.3 °C. Similar trends were observed in Layers 2 and 3, with the cooling effect generally becoming more pronounced at higher SAP replacement ratios. The results confirm that steel-encapsulated SAP–water aggregates effectively delay and attenuate the peak of hydration heat release while simultaneously reducing the cross-sectional temperature gradient, thereby mitigating potential thermal stress concentration.

The simulated internal peak temperatures and the maximum temperature differences between the interior and exterior surfaces for each casting layer are listed in Table 6. The inner–outer temperature difference was used as the main indicator for comparing the thermal gradient tendency among different mixtures under the same boundary and curing conditions. In practical mass concrete structures, the absolute temperature gradient is strongly affected by curing conditions, insulation, ambient temperature, wind speed, casting sequence, and cooling measures. Therefore, this study focused on the relative reduction in core temperature and inner–outer temperature difference rather than using the model to provide a direct construction-design temperature-gradient criterion. Compared with SAP-0, the SAP-modified mixtures generally reduced the internal peak temperature and the inner–outer temperature difference, indicating a lower tendency for thermal-gradient-driven stress development.

Table 7 presents the simulated thermal stress values at 3 and 7 days for the selected monitoring points. In this study, negative values represent compressive stress, while positive values represent tensile stress. The simulation shows that the central regions of the pile cap were mainly under compression, whereas the near-surface regions were mainly under tension, which is consistent with the typical thermal stress distribution in mass concrete [45,46,47]. After the incorporation of steel-encapsulated SAP–water aggregates, the absolute stress values generally decreased. For example, at the center of Layer 1, the compressive stress decreased from −11.91 MPa for SAP-0 to −10.68 MPa for SAP-15 at 3 days, and from −9.01 MPa to −8.24 MPa at 7 days. A similar but relatively moderate reduction was observed for the tensile stresses in the near-surface regions. These stress changes are mainly associated with the reduced core temperature and smaller inner–outer temperature difference caused by the thermal buffering effect of the SAP–water aggregates.

Through combined experimental and numerical analysis, this study shows that steel-encapsulated SAP–water aggregates can reduce the peak adiabatic temperature rise and delay the occurrence of the temperature peak. Under the same modeled boundary and curing conditions, the SAP-modified concretes exhibited lower internal peak temperatures, smaller inner–outer temperature differences, and reduced simulated thermal stresses. However, the finite element analysis was intended to compare the relative temperature-control effects of different aggregate replacement ratios, rather than to provide direct construction guidance or a complete cracking-risk assessment for a specific project. Therefore, the engineering-scale simulation should be interpreted as a comparative evaluation of the thermal-regulation potential of the proposed aggregates. Further studies incorporating age-dependent mechanical properties, actual curing conditions, and cracking-risk criteria are needed before direct engineering design applications.

## 5. Conclusions

This study developed a temperature-rise suppression concrete by partially replacing conventional coarse aggregate with steel-encapsulated SAP–water phase-change aggregates and evaluated its early-age thermal behavior through semi-adiabatic testing, adiabatic temperature-rise prediction, and finite element simulation. The main conclusions are as follows:

1. The incorporation of steel-encapsulated SAP–water phase-change aggregates reduced early-age heat accumulation under semi-adiabatic conditions, leading to both a lower temperature rise and a delayed thermal peak. The strongest effect was observed for SAP-15, in which the peak relative temperature rise decreased from 31.86 °C to 30.40 °C, and the peak time was delayed by 15.13 h.
2. Heat-loss-compensated analysis further confirmed the intrinsic thermal-buffering effect of the proposed aggregate system under equivalent adiabatic conditions. With increasing replacement ratio, the maximum adiabatic temperature rise decreased, and the heat-release history became flatter and more delayed. For SAP-15, the maximum adiabatic temperature rise decreased to 40.5 °C, while the specific heat capacity increased to 0.912 kJ/(kg·K).
3. The finite element model calibrated against the semi-adiabatic test results reproduced the measured temperature histories with good agreement, supporting the reliability of the proposed thermal analysis framework. The simulated peak core temperature decreased from 51 °C for SAP-0 to 44 °C for SAP-15.
4. Engineering-scale simulation of a bridge pile-cap foundation indicated that the laboratory-scale thermal benefit can be translated to mass concrete conditions. For SAP-15, the internal peak temperatures of the three casting layers were reduced by 5.3, 5.6, and 5.2 °C, respectively, relative to the reference concrete, together with a reduction in the temperature difference between the interior and exterior regions.
5. Overall, steel-encapsulated SAP–water phase-change aggregates show potential as a material-based strategy for moderating early-age temperature rise and simulated thermal stress in mass concrete. Further evaluation considering age-dependent mechanical properties, actual curing conditions, and cracking-risk criteria is required before direct engineering design application.

## Figures and Tables

**Table 1:** Chemical composition of the cementitious materials (wt%).

	CaO	Al_2_O_3_	SiO_2_	P_2_O_5_	MgO	SO_3_	K_2_O
P•O 42.5	63.07	7.42	21.51	0.12	1.29	2.25	0.94

**Table 2:** Mass stability test of the steel-encapsulated SAP–water aggregates after mortar mixing.

Test Condition	Number of Aggregates	Mean Mass Before Mixing (g)	Mean Mass After Mixing (g)	Mass Change Rate (%)	Observation
Mixed in cement mortar with w/c = 0.47 for 2 min	15	42.21	42.13	0.18	No visible leakage

**Table 3:** Mix proportions of the temperature-rise suppression concrete (kg/m^3^).

Number	Water-Cement Ratio	Water (kg/m^3^)	Cement (kg/m^3^)	Fine Aggregate (kg/m^3^)	Coarse Aggregate (kg/m^3^)	SAP–Water Phase-Change Aggregate (kg/m^3^)	Superplasticizer (kg/m^3^)
SAP-0	0.47	173	368	744	1115	0	0.7
SAP-5	0.47	173	368	744	1059.25	61.27	0.7
SAP-10	0.47	173	368	744	1003.5	122.55	0.7
SAP-15	0.47	173	368	744	947.75	183.82	0.7

**Table 4:** Key thermal parameters used for FEM heat-generation input.

Mixture	Density (kg/m^3^)	Thermal Conductivity, (W/(m·K))	Specific Heat Capacity, c (kJ/(kg·K))	Reaction Factor, *r* (h^−1^)	Maximum Adiabatic Temperature Rise, (Q_∞_) (°C)
SAP-0	2409.28	1.340	0.801	0.0615	46.7
SAP-5	2411.36	1.745	0.826	0.0608	44.2
SAP-10	2422.62	1.650	0.856	0.0603	44.1
SAP-15	2437.53	1.353	0.912	0.0598	40.5

Note: $Q_{\infty }$ and r were used to define the time-dependent hydration heat-generation function in the finite element model. The parameters were calibrated by minimizing the difference between the measured and simulated semi-adiabatic temperature histories.

**Table 5:** Thermophysical parameters and 28-day elastic modulus of the temperature-rise suppression concrete.

	Density (kg/m^3^)	Thermal Conductivity (W/(m·K))	Specific Heat Capacity (kJ/(kg·K))	Maximum Temperature Rise (°C)	Elastic Modulus (GPa)
SAP-0	2409.28	1.340	0.801	46.7	42.55
SAP-5	2411.36	1.745	0.826	44.2	37.8
SAP-10	2422.62	1.650	0.856	44.1	37.1
SAP-15	2437.53	1.353	0.912	40.5	38.4

Note: Thermal conductivity was measured at 25 °C using a TPS2500S thermal constant analyzer based on the Hot Disk transient plane source method, following ISO 22007-2. The reported thermal conductivity values are the averages of three repeated measurements. The elastic modulus values are the average results of three specimens tested at 28 days. In the finite element model, each mixture was treated as a homogenized concrete material, and the influence of the steel shells and SAP–water inclusions was reflected by the measured macroscopic elastic modulus.

**Table 6:** Simulated peak temperature and maximum inner–outer temperature difference for each casting layer.

	Casting Layer	Internal Peak Temperature (°C)			Max. Temp. Diff. (Inner–Outer) (°C)		
		Temperature (°C)	Reduction (°C)	Reduction Ratio (%)	Temperature (°C)	Reduction (°C)	Reduction Ratio (%)
SAP-0	Layer 1	69	/	/	9.9	/	/
SAP-5		66.9	2.1	3.04	8.7	1.2	12.12
SAP-10		66.7	2.3	3.33	8.0	1.9	19.19
SAP-15		63.7	5.3	7.68	8.6	1.3	13.13
SAP-0	Layer 2	69.3	/	/	9.3	/	/
SAP-5		67.1	2.2	3.17	8.9	0.4	4.3
SAP-10		67	2.3	3.32	8.9	0.4	4.3
SAP-15		63.7	5.6	8.08	7.9	1.4	15.05
SAP-0	Layer 3	64.8	/	/	7.8	/	/
SAP-5		62.6	2.2	3.4	7	0.8	10.26
SAP-10		62.6	2.2	3.4	6.9	0.9	11.54
SAP-15		59.6	5.2	8.02	7.1	0.7	8.97

**Table 7:** Simulated thermal stresses at selected locations in the mass concrete at 3 and 7 days.

	**3-Day Temperature Stress (MPa)**					
	**A1**	**A2**	**B1**	**B2**	**C1**	**C2**
SAP-0	−11.91	1.84	−10.55	1.44	−11.36	1.66
SAP-5	−11.49	1.82	−10.25	1.44	−10.84	1.63
SAP-10	−11.55	1.86	−10.38	1.49	−10.75	1.64
SAP-15	−10.68	1.74	−9.67	1.42	−9.80	1.50
	**7-Day Temperature Stress (MPa)**					
	**A1**	**A2**	**B1**	**B2**	**C1**	**C2**
SAP-0	−9.01	1.05	−7.38	0.93	−8.41	0.94
SAP-5	−8.73	1.03	−7.17	0.91	−8.18	0.92
SAP-10	−8.82	1.04	−7.27	0.92	−8.28	0.93
SAP-15	−8.24	0.99	−6.79	0.87	−7.76	0.88

Note: Negative values represent compressive stress, while positive values represent tensile stress. The simulated stresses are used for comparative evaluation of the relative stress-mitigation effect under the same boundary and curing conditions.

## Data Availability

The data supporting the findings of this study are available from the corresponding author upon reasonable request.

## Abbreviations

The following abbreviations are used in this manuscript:

SAP	Superabsorbent polymer
PCM	Phase-change material
FEA	Finite element analysis
EPS	Expanded polystyrene
SSD	Saturated-surface-dry
FEM	Finite element method
